# (4*R*,5*R*,10*S*)*-N*-(4-Bromo­phen­yl)dehydro­abietamide

**DOI:** 10.1107/S1600536809049575

**Published:** 2009-11-28

**Authors:** Wen Gu, Shifa Wang

**Affiliations:** a College of Chemical Engineering, Nanjing Forestry University, Nanjing 210037, People’s Republic of China

## Abstract

The title compound, C_26_H_32_BrNO, the ring with the amide unit possesses a chair conformation with the two methyl groups in axial positions..

## Related literature

For the synthesis and biological activity of dehydro­abietamide derivatives, see: Ntokos *et al.* (1973 ▶); Sepulveda *et al.* (2005 ▶); Fujita *et al.* (1991 ▶). For related structures see: Rao *et al.* (2006 ▶, 2007 ▶).
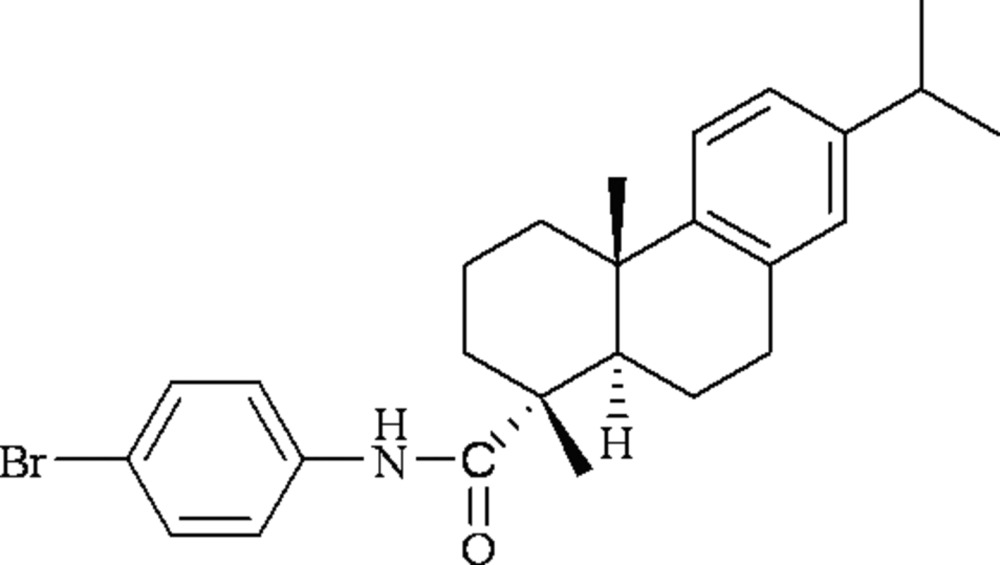


## Experimental

### 

#### Crystal data

C_26_H_32_BrNO
*M* *_r_* = 454.44
Orthorhombic, 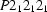

*a* = 5.9640 (12) Å
*b* = 11.750 (2) Å
*c* = 32.758 (7) Å
*V* = 2295.6 (8) Å^3^
*Z* = 4
Mo *K*α radiation
μ = 1.81 mm^−1^
*T* = 293 K
0.20 × 0.10 × 0.10 mm

#### Data collection

Enraf–Nonius CAD-4 diffractometer
Absorption correction: ψ scan (North *et al.*, 1968 ▶) *T*_min_ = 0.714, *T*_max_ = 0.840
4770 measured reflections
4143 independent reflections
1931 reflections with *I* > 2σ(*I*)
*R*_int_ = 0.063
3 standard reflections every 200 reflections intensity decay: 1%

#### Refinement

*R*[*F* ^2^ > 2σ(*F* ^2^)] = 0.065
*wR*(*F* ^2^) = 0.133
*S* = 1.00
4143 reflections
257 parameters
H-atom parameters constrained
Δρ_max_ = 0.32 e Å^−3^
Δρ_min_ = −0.34 e Å^−3^
Absolute structure: Flack (1983 ▶), 1699 Friedel pairs
Flack parameter: −0.003 (16)

### 

Data collection: *CAD-4 EXPRESS* (Enraf–Nonius, 1989 ▶); cell refinement: *CAD-4 EXPRESS*; data reduction: *XCAD4* (Harms & Wocadlo,1995 ▶); program(s) used to solve structure: *SHELXS97* (Sheldrick, 2008 ▶); program(s) used to refine structure: *SHELXL97* (Sheldrick, 2008 ▶); molecular graphics: *SHELXTL* (Sheldrick, 2008 ▶); software used to prepare material for publication: *SHELXL97*.

## Supplementary Material

Crystal structure: contains datablocks I, global. DOI: 10.1107/S1600536809049575/ng2678sup1.cif

Structure factors: contains datablocks I. DOI: 10.1107/S1600536809049575/ng2678Isup2.hkl

Additional supplementary materials:  crystallographic information; 3D view; checkCIF report

## Figures and Tables

**Fig. 1.**
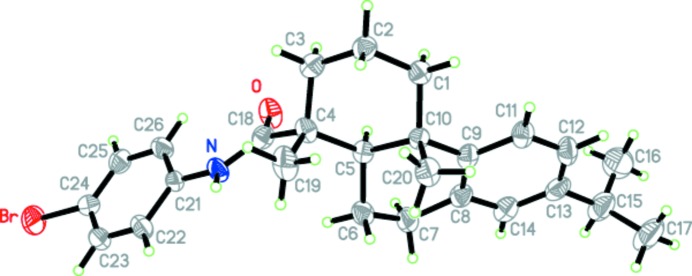
Molecular structure with 30% probability displacement ellipsoids for non-H atoms.

## supplementary crystallographic information

### Comment

As a part of our ongoing research on the synthesis and biological activities of dehydroabietic acid derivatives, a serial of dehydroabietamides were synthesized and the crystal structure of the title compound was detected.

### Experimental

To a solution of dehydroabietic acid (6.0 g, 0.02 mol) in benzene (40 ml) was added dropwise 2.16 ml of SOCl_2_ (3.6 g, 0.03 mol), and the mixture was refluxed for 3 h. After cooling to room temperature, the solvent was removed *in vacuo*, and the residue was then dissolved in 30 ml of benzene, to which triethylamine (2.02 g, 0.02 mol) and *p*-bromoaniline (3.6 g, 0.021 mol) were added, and the mixture was stirred at room temperature for 24 h. The mixture was then filtered to remove precipitate, the filtrate was evaporated *in vacuo* to afford a yellowish solid, which was recrystalized in EtOH to give (I) as colorless needles (7.3 g, 81%). Single crystals of (I) suitable for an X-ray diffraction study were obtained by slow evaporation of an acetone solution at room temperature over a period of 5 d.

### Refinement

All H atoms were placed geometrically with C—H = 0.93–0.98 Å, N—H = 0.86 Å and included in the refinement in riding motion approximation with *U*_iso_(H) = 1.2*U*_eq_ of the carrier atom.

### Crystal data

**Table:**

C_26_H_32_BrNO	*D*_x_ = 1.315 Mg m^−^^3^
*M**_r_* = 454.44	Mo *K*α radiation, λ = 0.71073 Å
Orthorhombic, *P*2_1_2_1_2_1_	Cell parameters from 25 reflections
*a* = 5.9640 (12) Å	θ = 10–13°
*b* = 11.750 (2) Å	µ = 1.81 mm^−^^1^
*c* = 32.758 (7) Å	*T* = 293 K
*V* = 2295.6 (8) Å^3^	Block, colourless
*Z* = 4	0.20 × 0.10 × 0.10 mm
*F*(000) = 952	

### Data collection

**Table:**

Enraf–Nonius CAD-4 diffractometer	1931 reflections with *I* > 2σ(*I*)
Radiation source: fine-focus sealed tube	*R*_int_ = 0.063
graphite	θ_max_ = 25.3°, θ_min_ = 1.2°
ω/2θ scans	*h* = 0→7
Absorption correction: ψ scan (North *et al.*, 1968)	*k* = 0→14
*T*_min_ = 0.714, *T*_max_ = 0.840	*l* = −39→39
4770 measured reflections	3 standard reflections every 200 reflections
4143 independent reflections	intensity decay: 1%

### Refinement

**Table:**

Refinement on *F*^2^	Secondary atom site location: difference Fourier map
Least-squares matrix: full	Hydrogen site location: inferred from neighbouring sites
*R*[*F*^2^ > 2σ(*F*^2^)] = 0.065	H-atom parameters constrained
*wR*(*F*^2^) = 0.133	*w* = 1/[σ^2^(*F*_o_^2^) + (0.048*P*)^2^] where *P* = (*F*_o_^2^ + 2*F*_c_^2^)/3
*S* = 1.00	(Δ/σ)_max_ < 0.001
4143 reflections	Δρ_max_ = 0.32 e Å^−^^3^
257 parameters	Δρ_min_ = −0.34 e Å^−^^3^
0 restraints	Absolute structure: Flack (1983), 1699 Friedel pairs
Primary atom site location: structure-invariant direct methods	Flack parameter: −0.003 (16)

### Special details

**Table:**

Geometry. All e.s.d.'s (except the e.s.d. in the dihedral angle between two l.s. planes) are estimated using the full covariance matrix. The cell e.s.d.'s are taken into account individually in the estimation of e.s.d.'s in distances, angles and torsion angles; correlations between e.s.d.'s in cell parameters are only used when they are defined by crystal symmetry. An approximate (isotropic) treatment of cell e.s.d.'s is used for estimating e.s.d.'s involving l.s. planes.
Refinement. Refinement of *F*^2^ against ALL reflections. The weighted *R*-factor *wR* and goodness of fit *S* are based on *F*^2^, conventional *R*-factors *R* are based on *F*, with *F* set to zero for negative *F*^2^. The threshold expression of *F*^2^ > σ(*F*^2^) is used only for calculating *R*-factors(gt) *etc*. and is not relevant to the choice of reflections for refinement. *R*-factors based on *F*^2^ are statistically about twice as large as those based on *F*, and *R*- factors based on ALL data will be even larger.

### Fractional atomic coordinates and isotropic or equivalent isotropic displacement parameters (Å^2^)

**Table:**

	*x*	*y*	*z*	*U*_iso_*/*U*_eq_	
Br	0.81958 (18)	0.68193 (7)	0.09535 (2)	0.0947 (4)	
N	0.8080 (10)	1.0580 (5)	−0.03060 (15)	0.0532 (14)	
H0A	0.9407	1.0744	−0.0390	0.064*	
O	0.4404 (8)	1.1045 (4)	−0.03859 (15)	0.0639 (14)	
C1	−0.1942 (15)	1.1802 (8)	−0.2890 (2)	0.109	
H1A	−0.2768	1.1527	−0.3121	0.164*	
H1B	−0.1996	1.2618	−0.2885	0.164*	
H1C	−0.2592	1.1506	−0.2644	0.164*	
C2	0.1482 (15)	1.1731 (9)	−0.3320 (2)	0.122 (4)	
H2A	0.0551	1.1471	−0.3540	0.183*	
H2B	0.2929	1.1377	−0.3339	0.183*	
H2C	0.1650	1.2542	−0.3337	0.183*	
C3	0.0417 (14)	1.1424 (8)	−0.2921 (2)	0.094 (3)	
H3A	0.0389	1.0591	−0.2910	0.112*	
C4	0.1823 (13)	1.1816 (8)	−0.2558 (2)	0.069 (2)	
C5	0.2310 (12)	1.2951 (8)	−0.2487 (2)	0.067 (2)	
H5A	0.1777	1.3507	−0.2665	0.081*	
C6	0.3610 (11)	1.3266 (6)	−0.21477 (18)	0.0565 (18)	
H6A	0.3951	1.4029	−0.2105	0.068*	
C7	0.4407 (11)	1.2442 (6)	−0.18691 (19)	0.0487 (18)	
C8	0.3956 (12)	1.1298 (6)	−0.1947 (2)	0.055 (2)	
C9	0.2662 (13)	1.1021 (7)	−0.2288 (2)	0.064 (2)	
H9A	0.2345	1.0258	−0.2335	0.077*	
C10	0.5908 (10)	1.2836 (5)	−0.15146 (18)	0.0399 (17)	
C11	0.5900 (9)	1.1879 (5)	−0.11856 (17)	0.0398 (15)	
H11A	0.4317	1.1781	−0.1114	0.048*	
C12	0.6589 (13)	1.0742 (5)	−0.13792 (19)	0.0555 (19)	
H12A	0.7988	1.0831	−0.1527	0.067*	
H12B	0.6804	1.0172	−0.1169	0.067*	
C13	0.4725 (14)	1.0366 (6)	−0.1672 (2)	0.068 (2)	
H13A	0.5267	0.9738	−0.1837	0.081*	
H13B	0.3459	1.0093	−0.1514	0.081*	
C14	0.4971 (11)	1.3919 (5)	−0.13126 (18)	0.0486 (18)	
H14A	0.5092	1.4546	−0.1504	0.058*	
H14B	0.3393	1.3806	−0.1254	0.058*	
C15	0.6185 (12)	1.4242 (5)	−0.09160 (19)	0.0575 (19)	
H15A	0.7739	1.4421	−0.0975	0.069*	
H15B	0.5491	1.4913	−0.0799	0.069*	
C16	0.6080 (11)	1.3265 (6)	−0.06107 (18)	0.0535 (18)	
H16A	0.4527	1.3132	−0.0537	0.064*	
H16B	0.6879	1.3483	−0.0365	0.064*	
C17	0.7083 (11)	1.2159 (5)	−0.0775 (2)	0.0470 (18)	
C18	0.8244 (11)	1.3108 (6)	−0.17050 (17)	0.0611 (18)	
H18A	0.8041	1.3499	−0.1960	0.092*	
H18B	0.9045	1.2411	−0.1752	0.092*	
H18C	0.9083	1.3580	−0.1521	0.092*	
C19	0.9710 (10)	1.2251 (6)	−0.0809 (2)	0.073 (3)	
H19A	1.0095	1.2840	−0.0999	0.110*	
H19B	1.0310	1.1538	−0.0902	0.110*	
H19C	1.0327	1.2429	−0.0546	0.110*	
C20	0.6416 (14)	1.1207 (6)	−0.04718 (19)	0.0519 (18)	
C21	0.7971 (13)	0.9705 (5)	−0.00191 (19)	0.0477 (17)	
C22	0.9833 (12)	0.9004 (5)	0.0016 (2)	0.0580 (19)	
H22A	1.1067	0.9120	−0.0153	0.070*	
C23	0.9861 (13)	0.8142 (7)	0.0298 (2)	0.063 (2)	
H23A	1.1096	0.7659	0.0311	0.075*	
C24	0.8111 (16)	0.7978 (6)	0.05601 (19)	0.061 (2)	
C25	0.6259 (13)	0.8674 (6)	0.05292 (19)	0.060 (2)	
H25A	0.5046	0.8560	0.0703	0.072*	
C26	0.6176 (12)	0.9541 (6)	0.02438 (19)	0.058 (2)	
H26A	0.4924	1.0010	0.0228	0.069*	

### Atomic displacement parameters (Å^2^)

**Table:**

	*U*^11^	*U*^22^	*U*^33^	*U*^12^	*U*^13^	*U*^23^
Br	0.1378 (8)	0.0749 (5)	0.0715 (5)	0.0229 (6)	−0.0134 (6)	0.0208 (5)
N	0.032 (3)	0.072 (4)	0.055 (3)	0.014 (4)	0.001 (3)	0.012 (3)
O	0.032 (3)	0.089 (4)	0.070 (3)	−0.006 (3)	0.002 (3)	0.028 (3)
C1	0.109	0.109	0.109	0.000	0.000	0.000
C2	0.093 (7)	0.220 (11)	0.054 (5)	0.027 (10)	−0.007 (6)	−0.031 (7)
C3	0.084 (7)	0.140 (9)	0.057 (5)	0.005 (6)	−0.012 (5)	−0.021 (5)
C4	0.059 (5)	0.100 (6)	0.048 (4)	0.005 (7)	0.013 (4)	−0.013 (5)
C5	0.048 (5)	0.102 (7)	0.051 (4)	0.010 (5)	−0.004 (4)	0.019 (5)
C6	0.047 (5)	0.073 (5)	0.049 (4)	−0.007 (5)	0.005 (4)	0.010 (4)
C7	0.034 (4)	0.071 (5)	0.041 (4)	−0.004 (4)	0.008 (4)	0.010 (4)
C8	0.052 (5)	0.063 (5)	0.050 (4)	−0.003 (4)	−0.006 (4)	−0.009 (4)
C9	0.062 (6)	0.078 (5)	0.053 (4)	−0.007 (4)	0.001 (4)	−0.007 (4)
C10	0.027 (4)	0.054 (4)	0.039 (4)	−0.001 (3)	0.004 (3)	0.001 (3)
C11	0.019 (3)	0.055 (4)	0.045 (3)	−0.010 (4)	0.000 (3)	0.004 (4)
C12	0.060 (5)	0.049 (4)	0.058 (4)	0.001 (5)	0.003 (5)	0.012 (3)
C13	0.092 (6)	0.053 (5)	0.058 (5)	0.009 (5)	−0.011 (5)	0.005 (4)
C14	0.046 (4)	0.051 (4)	0.050 (4)	−0.004 (4)	0.006 (4)	0.007 (3)
C15	0.066 (5)	0.053 (4)	0.053 (4)	−0.003 (4)	−0.003 (5)	−0.003 (4)
C16	0.045 (5)	0.064 (4)	0.052 (4)	−0.008 (4)	0.000 (3)	−0.007 (4)
C17	0.027 (4)	0.057 (4)	0.057 (4)	−0.004 (4)	0.001 (4)	0.012 (3)
C18	0.042 (4)	0.082 (5)	0.059 (4)	−0.003 (5)	0.015 (4)	0.021 (4)
C19	0.028 (4)	0.113 (7)	0.079 (6)	−0.012 (5)	−0.011 (4)	0.018 (5)
C20	0.050 (5)	0.060 (4)	0.046 (4)	−0.005 (5)	−0.003 (4)	−0.004 (3)
C21	0.047 (4)	0.051 (4)	0.045 (4)	0.011 (4)	−0.015 (4)	−0.006 (3)
C22	0.052 (5)	0.057 (5)	0.066 (5)	0.009 (4)	−0.010 (4)	0.003 (4)
C23	0.065 (6)	0.058 (5)	0.064 (5)	0.017 (5)	−0.015 (4)	−0.006 (5)
C24	0.071 (5)	0.058 (5)	0.054 (4)	0.014 (5)	−0.005 (5)	−0.009 (4)
C25	0.068 (6)	0.064 (5)	0.047 (4)	−0.005 (5)	0.005 (4)	0.005 (4)
C26	0.051 (5)	0.065 (5)	0.057 (5)	0.013 (4)	0.006 (4)	0.006 (4)

### Geometric parameters (Å, °)

**Table:**

Br—C24	1.876 (7)	C12—C13	1.534 (9)
N—C20	1.350 (8)	C12—H12A	0.9700
N—C21	1.394 (7)	C12—H12B	0.9700
N—H0A	0.8600	C13—H13A	0.9700
O—C20	1.247 (7)	C13—H13B	0.9700
C1—C3	1.478 (11)	C14—C15	1.535 (8)
C1—H1A	0.9600	C14—H14A	0.9700
C1—H1B	0.9600	C14—H14B	0.9700
C1—H1C	0.9600	C15—C16	1.524 (8)
C2—C3	1.498 (10)	C15—H15A	0.9700
C2—H2A	0.9600	C15—H15B	0.9700
C2—H2B	0.9600	C16—C17	1.528 (9)
C2—H2C	0.9600	C16—H16A	0.9700
C3—C4	1.525 (10)	C16—H16B	0.9700
C3—H3A	0.9800	C17—C20	1.548 (8)
C4—C9	1.382 (10)	C17—C19	1.574 (8)
C4—C5	1.385 (10)	C18—H18A	0.9600
C5—C6	1.406 (9)	C18—H18B	0.9600
C5—H5A	0.9300	C18—H18C	0.9600
C6—C7	1.413 (8)	C19—H19A	0.9600
C6—H6A	0.9300	C19—H19B	0.9600
C7—C8	1.394 (8)	C19—H19C	0.9600
C7—C10	1.538 (9)	C21—C22	1.387 (8)
C8—C9	1.396 (8)	C21—C26	1.388 (8)
C8—C13	1.489 (9)	C22—C23	1.372 (9)
C9—H9A	0.9300	C22—H22A	0.9300
C10—C14	1.540 (8)	C23—C24	1.365 (9)
C10—C11	1.557 (8)	C23—H23A	0.9300
C10—C18	1.559 (8)	C24—C25	1.378 (9)
C11—C12	1.535 (8)	C25—C26	1.383 (8)
C11—C17	1.555 (8)	C25—H25A	0.9300
C11—H11A	0.9800	C26—H26A	0.9300
			
C20—N—C21	129.7 (6)	C8—C13—H13B	109.0
C20—N—H0A	115.1	C12—C13—H13B	109.0
C21—N—H0A	115.1	H13A—C13—H13B	107.8
C3—C1—H1A	109.5	C15—C14—C10	113.4 (5)
C3—C1—H1B	109.5	C15—C14—H14A	108.9
H1A—C1—H1B	109.5	C10—C14—H14A	108.9
C3—C1—H1C	109.5	C15—C14—H14B	108.9
H1A—C1—H1C	109.5	C10—C14—H14B	108.9
H1B—C1—H1C	109.5	H14A—C14—H14B	107.7
C3—C2—H2A	109.5	C16—C15—C14	110.5 (5)
C3—C2—H2B	109.5	C16—C15—H15A	109.6
H2A—C2—H2B	109.5	C14—C15—H15A	109.6
C3—C2—H2C	109.5	C16—C15—H15B	109.6
H2A—C2—H2C	109.5	C14—C15—H15B	109.6
H2B—C2—H2C	109.5	H15A—C15—H15B	108.1
C1—C3—C2	113.0 (8)	C15—C16—C17	113.1 (5)
C1—C3—C4	112.3 (7)	C15—C16—H16A	108.9
C2—C3—C4	112.0 (7)	C17—C16—H16A	108.9
C1—C3—H3A	106.3	C15—C16—H16B	108.9
C2—C3—H3A	106.3	C17—C16—H16B	108.9
C4—C3—H3A	106.3	H16A—C16—H16B	107.8
C9—C4—C5	117.9 (7)	C16—C17—C20	106.7 (5)
C9—C4—C3	119.7 (8)	C16—C17—C11	107.9 (5)
C5—C4—C3	122.5 (8)	C20—C17—C11	106.6 (5)
C4—C5—C6	120.1 (7)	C16—C17—C19	110.9 (6)
C4—C5—H5A	119.9	C20—C17—C19	110.5 (6)
C6—C5—H5A	119.9	C11—C17—C19	113.9 (5)
C5—C6—C7	121.1 (7)	C10—C18—H18A	109.5
C5—C6—H6A	119.4	C10—C18—H18B	109.5
C7—C6—H6A	119.4	H18A—C18—H18B	109.5
C8—C7—C6	118.6 (6)	C10—C18—H18C	109.5
C8—C7—C10	122.7 (6)	H18A—C18—H18C	109.5
C6—C7—C10	118.5 (6)	H18B—C18—H18C	109.5
C7—C8—C9	118.5 (7)	C17—C19—H19A	109.5
C7—C8—C13	122.7 (6)	C17—C19—H19B	109.5
C9—C8—C13	118.8 (7)	H19A—C19—H19B	109.5
C4—C9—C8	123.8 (7)	C17—C19—H19C	109.5
C4—C9—H9A	118.1	H19A—C19—H19C	109.5
C8—C9—H9A	118.1	H19B—C19—H19C	109.5
C7—C10—C14	111.2 (5)	O—C20—N	122.2 (7)
C7—C10—C11	107.7 (5)	O—C20—C17	120.1 (7)
C14—C10—C11	107.3 (5)	N—C20—C17	117.6 (7)
C7—C10—C18	106.3 (5)	C22—C21—C26	118.9 (6)
C14—C10—C18	109.0 (5)	C22—C21—N	117.1 (7)
C11—C10—C18	115.3 (5)	C26—C21—N	123.9 (7)
C12—C11—C17	114.8 (5)	C23—C22—C21	120.2 (7)
C12—C11—C10	110.0 (5)	C23—C22—H22A	119.9
C17—C11—C10	116.4 (5)	C21—C22—H22A	119.9
C12—C11—H11A	104.7	C24—C23—C22	121.2 (7)
C17—C11—H11A	104.7	C24—C23—H23A	119.4
C10—C11—H11A	104.7	C22—C23—H23A	119.4
C13—C12—C11	108.4 (6)	C23—C24—C25	118.9 (6)
C13—C12—H12A	110.0	C23—C24—Br	120.9 (6)
C11—C12—H12A	110.0	C25—C24—Br	120.2 (6)
C13—C12—H12B	110.0	C24—C25—C26	121.0 (7)
C11—C12—H12B	110.0	C24—C25—H25A	119.5
H12A—C12—H12B	108.4	C26—C25—H25A	119.5
C8—C13—C12	112.9 (6)	C25—C26—C21	119.7 (7)
C8—C13—H13A	109.0	C25—C26—H26A	120.2
C12—C13—H13A	109.0	C21—C26—H26A	120.2
			
C1—C3—C4—C9	113.1 (9)	C7—C10—C14—C15	170.8 (5)
C2—C3—C4—C9	−118.5 (9)	C11—C10—C14—C15	53.2 (6)
C1—C3—C4—C5	−67.0 (11)	C18—C10—C14—C15	−72.3 (6)
C2—C3—C4—C5	61.4 (11)	C10—C14—C15—C16	−57.3 (7)
C9—C4—C5—C6	−0.3 (11)	C14—C15—C16—C17	57.7 (7)
C3—C4—C5—C6	179.8 (7)	C15—C16—C17—C20	−168.5 (6)
C4—C5—C6—C7	−1.2 (10)	C15—C16—C17—C11	−54.3 (7)
C5—C6—C7—C8	2.6 (10)	C15—C16—C17—C19	71.0 (7)
C5—C6—C7—C10	177.3 (6)	C12—C11—C17—C16	−175.8 (5)
C6—C7—C8—C9	−2.5 (10)	C10—C11—C17—C16	53.7 (7)
C10—C7—C8—C9	−176.9 (6)	C12—C11—C17—C20	−61.5 (7)
C6—C7—C8—C13	179.9 (6)	C10—C11—C17—C20	168.0 (5)
C10—C7—C8—C13	5.4 (11)	C12—C11—C17—C19	60.7 (7)
C5—C4—C9—C8	0.4 (11)	C10—C11—C17—C19	−69.9 (7)
C3—C4—C9—C8	−179.7 (7)	C21—N—C20—O	−1.7 (11)
C7—C8—C9—C4	1.0 (11)	C21—N—C20—C17	177.8 (6)
C13—C8—C9—C4	178.8 (7)	C16—C17—C20—O	55.3 (8)
C8—C7—C10—C14	−141.0 (6)	C11—C17—C20—O	−59.8 (8)
C6—C7—C10—C14	44.5 (7)	C19—C17—C20—O	176.0 (6)
C8—C7—C10—C11	−23.7 (8)	C16—C17—C20—N	−124.2 (6)
C6—C7—C10—C11	161.9 (5)	C11—C17—C20—N	120.7 (6)
C8—C7—C10—C18	100.4 (7)	C19—C17—C20—N	−3.5 (8)
C6—C7—C10—C18	−74.0 (8)	C20—N—C21—C22	162.9 (6)
C7—C10—C11—C12	54.4 (7)	C20—N—C21—C26	−20.7 (10)
C14—C10—C11—C12	174.2 (5)	C26—C21—C22—C23	1.9 (10)
C18—C10—C11—C12	−64.0 (7)	N—C21—C22—C23	178.6 (6)
C7—C10—C11—C17	−172.8 (5)	C21—C22—C23—C24	−2.5 (11)
C14—C10—C11—C17	−53.0 (7)	C22—C23—C24—C25	2.1 (11)
C18—C10—C11—C17	68.8 (7)	C22—C23—C24—Br	−178.5 (5)
C17—C11—C12—C13	158.1 (5)	C23—C24—C25—C26	−1.3 (11)
C10—C11—C12—C13	−68.2 (6)	Br—C24—C25—C26	179.3 (5)
C7—C8—C13—C12	−17.0 (10)	C24—C25—C26—C21	0.8 (10)
C9—C8—C13—C12	165.4 (6)	C22—C21—C26—C25	−1.1 (10)
C11—C12—C13—C8	47.0 (7)	N—C21—C26—C25	−177.5 (6)
